# The Significance of Interstitial Fibrosis on Left Ventricular Function in Hypertensive versus Hypertrophic Cardiomyopathy

**DOI:** 10.1038/s41598-018-27049-1

**Published:** 2018-07-03

**Authors:** Meng Jiang, Zi Wang, Xuan Su, Xingrong Gong, Jun Pu, Lianming Wu, Chang Liu, Qiuying Yao, Lingcong Kong, Jianrong Xu, Ben He

**Affiliations:** 1 Department of Cardiology, Renji Hospital, School of Medicine Shanghai Jiaotong University, Shanghai, 200127 China; 2 Department of Radiology, Renji Hospital, School of Medicine Shanghai Jiaotong University, Shanghai, 200127 China; 3 Department of Epidemiology and Biostatistics, West China School of Public Health, Sichuan University, Chengdu, 610041 China

## Abstract

Extracellular volume (ECV) has been validated as a surrogate measure of interstitial fibrosis, that is increased in both hypertension-induced left ventricular hypertrophy (H-LVH) and hypertrophic cardiomyopathy (HCM). We aimed to explore the correlation between ECV and left ventricular cardiac function. Eighty-one patients with HCM, 44 with H-LVH and 35 controls were prospectively enrolled. Even among patients with normal diastolic function, patients in HCM group had increased- ECV. In terms of diastolic dysfunction (DD), a similar increase in ECV was associated with a larger percentage of patients with severe or moderate-to-severe DD in HCM group. In addition, there was a compensatory increase in the left ventricular ejection fraction (LVEF) in HCM, but no hyperdynamic LVEF was observed in H-LVH. ECV was negatively correlated with LVEF in the late gadolinium enhancement (+) (LGE+) subgroups in the H-LVH group, while no significant linear correlation was observed in HCM group. The increased ECV in HCM patients with normal diastolic function warrants further exploration of the prognostic value of ECV assessments in the early stages of HCM. The associations between ECV and left ventricular functional parameters differed and taking both LGE and ECV into account might be reasonable way to differentiate between the two disorders.

## Introduction

Although both hypertension-induced left ventricular hypertrophy (H-LVH) and hypertrophic cardiomyopathy (HCM) have common features, such as familial heredity, myocardial hypertrophy and possibly diastolic dysfunction, the extent and pattern of these hypertrophies differ, perhaps due to underlying distinct pathophysiologies. Histologically, hypertensive cardiac remodelling is the result of adaptive changes to pressure overload^1^. However, the characteristics of HCM may be due to an intramural, congenital component of the underlying cardiomyopathy process^2^. Hypertrophic remodelling in both disorders has been documented to be associated with altered collagen synthesis and degradation and increased extracellular volume (ECV)^3,4^. Although increased ECV may be one of the mechanisms of altered cardiac function, whether the same magnitude of one increase in ECV is related to a similar degree of hypertrophy in these two disorders is unclear. More importantly, little is known about the correlation between ECV and systolic and diastolic function. The differences in the ECV that are responsible for altered systolic and diastolic function that lead to with regard to different causes of hypertrophy are not completely understood but could be important for treatment timing and treatment options in these disorders^5,6^.

ECV measured with cardiac magnetic resonance (CMR) imaging techniques, which use regions of interest drawn on CMR T1 maps, has been validated as a surrogate measure of interstitial fibrosis with high accuracy and reproducibility^7^. Several recent clinical studies have suggested that T1 mapping with ECV estimations will advance our understanding of the role of the extsracellular matrix in numerous pathophysiological processes of the human heart^8^. This technique has been especially useful in cases of homogenous “diffuse fibrosis,” such as in H-LVH^3,9^ and HCM^4^. Because the myocardial scarring visualized via late gadolinium enhancement (LGE) and the calculated ECV may not yield the same findings, the same degree of interstitial fibrosis in H-LVH and HCM patients may not be visualized equally on CMR. Therefore, when combining multi-modality imaging with the currently available T1 maps, ECV may provide insights into the relationships between visible and non-visible fibrosis, clinical features and pathological changes.

The goal of this study was to evaluate the relationship between ECV changes and diastolic and systolic functions in patients with H-LVH and HCM to better understand the underlying pathologies of these two cardiomyopathies.

## Results

### Patient Enrolment

One hundred sixty-seven patients were enrolled based on the inclusion and exclusion criteria. One patient did not complete post-contrast T1 mapping due to heart failure. Two patients scored a 1 on ECV mapping (1 patient with LVH and 1 patient with HCM) and were not included due to image artefacts that precluded analysis. Three were diagnosed with infiltrative cardiomyopathy and one was confirmed to have coronary artery disease after CMR. One hundred sixty patients were separated into 3 groups (35 in the control group, 44 in the H-LVH group and 81 in the HCM group).

### Assessment of the Reproducibility of ECV and T1 Values

A total of 960 maps corresponding to 160 consecutively imaged patients were scored. Of 5,120 segments, 5,018 (98%) segments were able to be analysed (score greater than or equal to 2). A Bland- Altman plot showed excellent agreement between operators for ECV, with average difference estimates of 0.002 (95% CI of 0.039) and 0.042 (r = 0.94, p < 0.001). The same reasonable agreement was observed for the native T1 (the average difference estimate was 1.59, 95% CI of −57.57 to 59.76; r = 0.93, p < 0.001) and post-T1 measurements (the average difference estimate was −0.98, 95% CI of −43.76 to 42.78; r = 0.96, p < 0.001).

### Patient Characteristics

The patient characteristics are shown in Table 1. Age, gender, diabetes status and hyperlipidaemia status were not different among the 3 groups (p > 0.05). Patients in the disease groups had higher blood pressure and lower estimated glomerular filtration rates than those of the controls (p < 0.001); therefore, they were more likely to receive medical treatment (p < 0.05). In terms of LV morphology, the maximum end-diastolic wall thickness was greater in the HCM group than in the H-LVH group (17.0 (14.5–20.0) mm in the HCM group vs. 13.5 (12.7**–**16.0) mm in the H-LVH group, p < 0.001), but the LVM and LVMI were similar between the H-LVH and HCM groups (p > 0.05). In terms of LV function, the LVEF was comparable between the H-LVH group and the control group, but it was hyper-dynamic in the HCM group (p = 0.7 for the H-LVH group vs. the control group; p = 0.001 for the HCM group vs. the control group). A higher LV mass/volume ratio was found in the H-LVH and HCM groups than in the control group (p < 0.001). The presence of scarring was found in only 15 of 44 (34.1%) patients with H-LVH, but it was frequently (68 of 81) found in the HCM group (84%) (p < 0.001). Visually recognizable scar sizes were much smaller in the H- LVH group (0**–**0.94% for the interquartile range) than in the HCM group (2.21**–**15.10% for the interquartile range; p < 0.001).

**Table 1:** Clinical characteristics.

	Control (n = 35)	H-LVH (n = 44)	HCM (n = 81)	P value*
Clinical				
Age (yrs)	52.8 ± 14.8	54.4 ± 15.3	55.4 ± 14.3	0.502
Male gender (%)	19 (54.3)	33 (75.0)	48 (59.3)	0.197
Risk factors				
Hypertension	0 (0)	44(100.0)	29 (42.0)^†^	**<0.001**
Diabetes	2 (5.7)	6 (13.6)	8 (9.9)	0.676
Current smoking	0 (0)	7 (15.9)	15 (18.5)	**0.022**
Hyperlipidaemia	4 (11.4)	14 (31.8)	16 (19.8)	0.064
NYHA class III/IV	—	2 (5)	0 (0)	0.122
Vital signs				
Heart rate, beats/min	71.2 ± 13.7	73.4 ± 13.7	69.4 ± 11.0	0.225
Systolic BP, mmHg	118 (110–125)	150 (131–160)	130 (120–140)^†^	**<0.001**
Diastolic BP, mmHg	74 (70–80)	90 (79–100)	76 (70–80)^†^	**<0.001**
Lab findings				
eGFR, ml/min/1.73 m^2^	115.0 ± 20.8	86.9 ± 33.4	97.9 ± 27.9	**<0.001**
Haematocrit, %	42.4 ± 3.5	40.4 ± 5.6	42.2 ± 4.8	0.073
Medications				
β-blocker	7 (20.0)	21 (47.7)	49 (60.5)	**<0.001**
ACEI or ARB	0 (0)	31 (70.5)	53 (65.4)	**<0.001**
CCB	0 (0)	25 (56.8)	27 (33.3)^†^	**<0.001**
Diuretics	0 (0)	21 (47.7)	11 (13.6)^†^	**<0.001**
CMR				
Morphology and Function				
Maximum EDWT, mm	7.8 (6.3–9.4)	13.5 (12.7–16.0)	17.0 (14.5–20.0)^†^	**<0.001**
LV mass, g	100.2 (76–116.8)	180.7 (155.3–229.5)	201.9 (145.7–255.1)	**<0.001**
LV mass index, g/m^2‡^	56.5 (46.8–62.4)	100.6 (84.1–121.8)	115 (85.46–143.19)	**0.033**
LVEF, %	65.6 (61.1–70.5)	66.13 (57.6–74.9)	76.1 (65.9–79.3)^†^	**<0.001**
LVEDV, ml	124.7 (104.7–140.6)	142.8 (110.6–183.8)	113.5 (98.1–134.4)^†^	**<0.001**
LVEDV index, ml/m^2‡^	70.1 (61.5–81.7)	77.2 (64.4–102.2)	63.17 (56.29–76.7)^†^	**0.004**
LVESV, ml	44.0 (35.1–52.7)	44.9 (32.23–67.5)	29.9 (21.05–38.65)^†^	**<0.001**
LVESV index, ml/m^2‡^	24.8 (18.8–29.4)	25.6 (17.0–38.2)	17.2 (11.8–22.0)^†^	**<0.001**
LV mass/volume ratio	0.81 (0.65–0.97)	1.33 (1.18–1.66)	1.72 (1.33–2.30)^†^	**<0.001**
Myocardial Scarring				
Scar	0	15 (34.1)	68 (84.0)^†^	**<0.001**
Scar size, %	0 (0)	0–0.94	2.21–15.10^†^	**<0.001**

Values are presented as the mean ± SD, number (%), or median (25th-75th percentile). Numbers in boldface indicate P values < 0.05.

^*^P value across the 3 groups.

^†^P < 0.05 between the H-LVH and HCM groups.

^‡^Indexed to the body surface area.

ACEI = angiotensin-converting-enzyme inhibitor; ARB = angiotensin II receptor blockers; BP = blood pressure; CCB = calcium channel blockers; CMR = cardiac magnetic resonance; EDWT = end-diastolic wall thickness; eGFR = glomerular filtration rate; HCM = hypertrophic cardiomyopathy; LV = left ventricle; LVEF = left ventricular ejection fraction; LVEDV = left ventricular end diastolic volume; LVESV = left ventricular end systolic volume; and NYHA = New York Heart Association.

### T1 Relaxation Times in the H-LVH HCM, and Control Groups

Table 2 summarizes the T1 relaxation times between groups. The mean T1 relaxation time tended to be increased for native T1 and decreased for post-contrast T1 in the disease groups compared with the control group (native T1: H-LVH group = 1,271 ± 81 ms, HCM group = 1,291 ± 69 ms and control group = 1,252 ± 49 ms, p < 0.001; post-contrast T1: H-LVH group = 587 ± 66 ms, HCM group = 584 ± 43 ms and control group = 623 ± 37 ms, p = 0.009). The ECV was greater in both cardiomyopathy groups than in the control group (28 ± 3% for the HCM group vs. 24 ± 2% for the control group, p < 0.001; 28 ± 4% for the H-LVH group vs. the control group, p < 0.001; and p = 0.8 for the HCM group vs. the H-LVH group).

**Table 2:** T1 Times and T1 Induced ECV.

	Control (n = 35)	H-LVH (n = 44)	HCM (n = 81)	P value*
Native T1				
T1 myocardium, ms	1252 ± 49	1271 ± 81	1291 ± 69	<**0**.**001**
T1 blood, ms	1785 ± 158	1801 ± 164	1819 ± 128	0.507
Post-contrast T1				
T1 myocardium, ms	623 ± 37	587 ± 66	584 ± 43	**0**.**009**
T1 blood, ms	397 ± 33	407 ± 81	400 ± 42	0.612
ECV (%)	24 ± 2	28 ± 4	28 ± 3	<**0**.**001**

Values were acquired on 3 Tesla Scanners. Values are presented as the mean ± SD, number (%). Numbers in boldface indicate P values < 0.05.

*P value across the 3 groups.

H-LVH = hypertension induced left ventricular hypertrophy; HCM = hypertrophic cardiomyopathy; and ECV = extracellular volume.

### Correlations between ECV and Myocardial Hypertrophy

**Figure 1:**
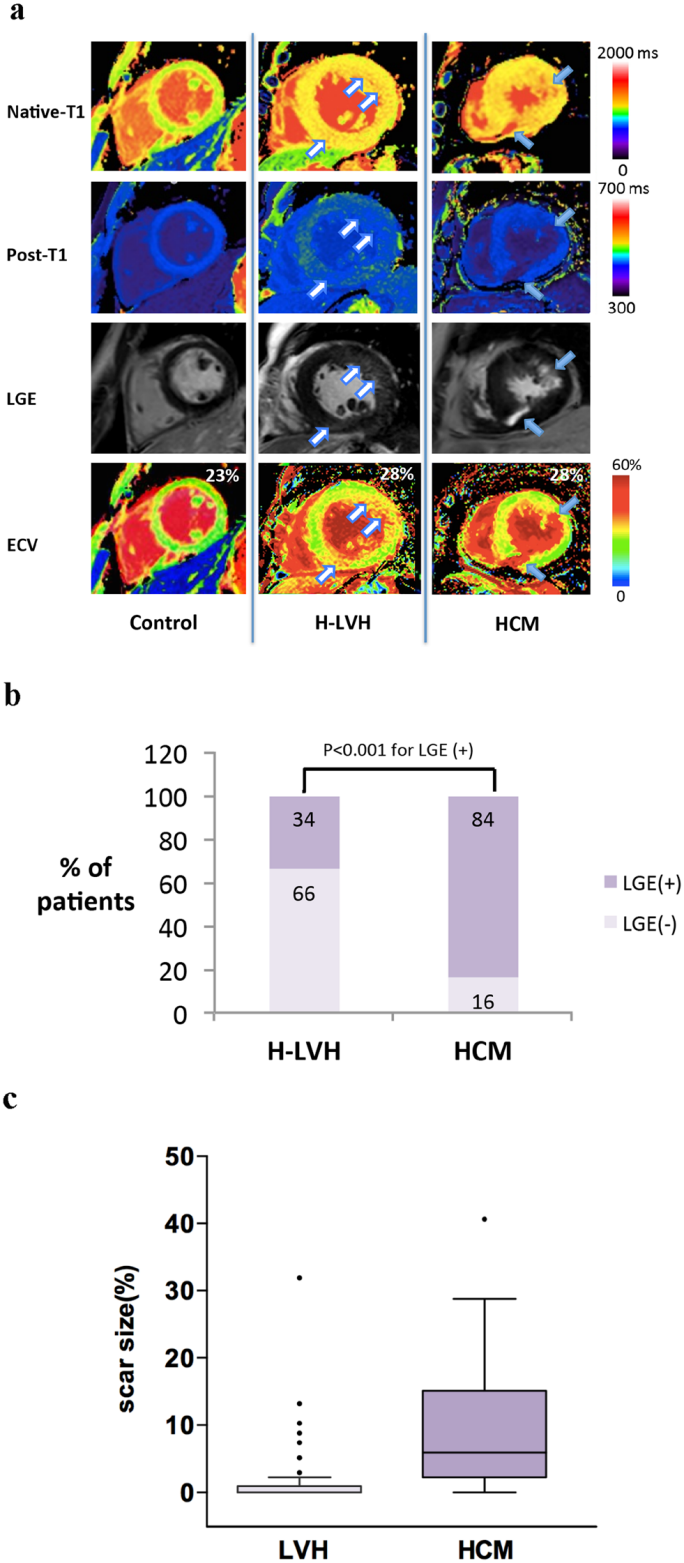
Fibrotic Distribution and Extent. (**a**) Examples illustrating myocardial T1 values and LGE characteristics in cases of abnormalities in H-LVH and HCM patients. Higher global native T1 values (yellow in cardiomyopathies, green in controls) and lower post-contrast T1 values (blue-purple in cardiomyopathies, light blue in controls) were seen in patients with H-LVH and HCM than in controls. Note the reddish colour on the native T1 and the blue-purple dots on the post-T1 images diffusively distributed in H-LVH patients (hollow arrows) compared to the much denser but concentrated foci in HCM patients; in HCM patients, the dense concentrated foci enabled the hyperenhancement that is visibly present with the conventional LGE technique (blue arrows). The same ECV (28%) was calculated for the H-LVH and HCM groups, which was higher than that of the control group (23%). (**b**–**c**) Presence and percentage of LGE. LGE was present in 84% of the patients with HCM and the interquartile range of the scar size was 2.2–15.1%. Conversely, only 34% of the H-LVH patients were LGE-positive, and the scar size ranged from 0–0.9%. ECV = extracellular volume; HCM = hypertrophic cardiomyopathy; H-LVH = hypertension-induced left ventricular hypertrophy; LGE = late gadolinium enhancement.

The same LVMI values corresponded to similar ECV values (coefficient = 0.02, standard error = 0.01 for both the H-LVH and HCM groups). The ECV did not differ based on the type of disease but showed distinct distributions between the two diseases (Fig. 1a). Patient examples of T1 colour mapping showed that although the same magnitude of increase in ECV (28%) was calculated for both groups (bottom panel); diffuse dots represent the increased ECV in the H-LVH group (hollow arrows) while much denser but concentrated foci are depicted in the HCM group (blue arrows). Almost no recognizable scarring was detected in the H-LVH group but was clearly evident in patients with HCM (lower middle panel). In terms of the visually recognizable LGE (Fig. 1b–c), 84% of the patients with HCM were LGE-positive and had scar sizes ranging from 0% to 40.6% (median 5.9% (inter-quartile range 2.2–15.1%)); LGE was infrequently seen (34% of the patients) and was seen to a lesser extent in patients with H-LVH than in patients with HCM (ranging from 0–31.9%, median 0%, inter-quartile range 0–0.9%).

### Impact of Fibrosis on Cardiac Function

**Figure 2:**
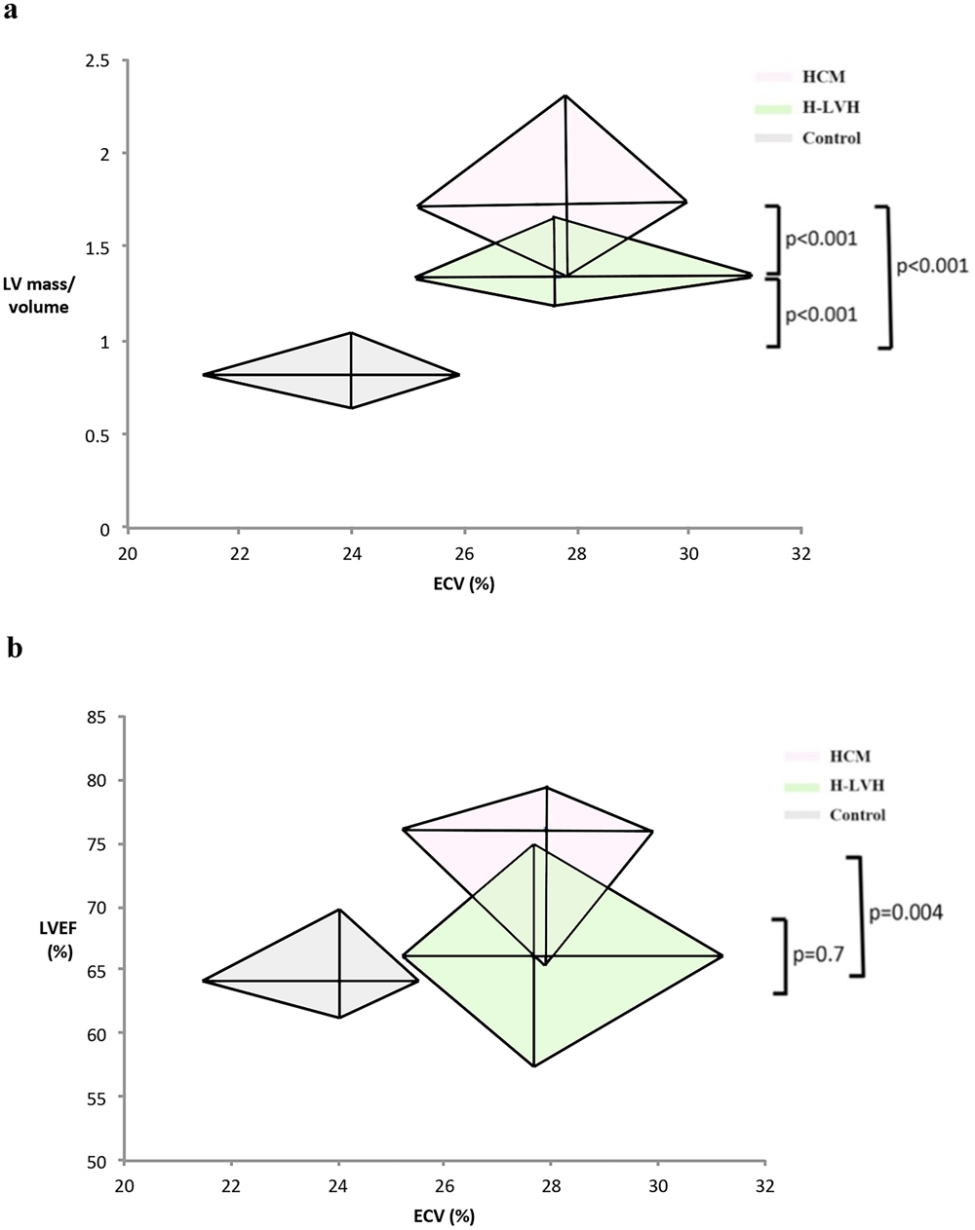
Relationship between ECV and Cardiac Function. (**a**) The relationship between ECV and the LV mass/volume ratio. With the same range of ECVs (p = 0.6 for the H-LVH group vs. the HCM group), the LV mass/volume ratio was mildly impaired in H-LVH patients (1.19–1.66 compared with 0.65–0.97 in controls, p < 0.001), but it was markedly impaired in HCM patients (1.33–2.30, p < 0.001 compared with H-LVH or controls). (**b**) Relationship between ECV and LVEF. A hyperdynamic LVEF was observed in HCM patients but was not observed in patients with H-LVH for correspondingly similar ranges of ECV (p = 0.004 for the HCM group vs. the control group and p = 0.7 for the H-LVH group vs. the control group). ECV: extracellular volume; H-LVH: hypertension-induced left ventricular hypertrophy; HCM: hypertrophic cardiomyopathy; LV: left ventricle; LVEF: left ventricular ejection fraction.

The impact of ECV on diastolic properties is shown in Tables 3–4 and Fig. 2a. Even in patients with normal diastolic function, patients with H-LVH or HCM had increased native T1 values (1,282 ± 64 in the H-LVH group vs. 1,288 ± 43 in the HCM group vs. 1,252 ± 49 in the control group, p = 0.03) and greater ECVs than the controls (25 ± 3% in the H-LVH group vs. 25 ± 2% in the HCM group vs. 24 ± 2% in the control group, p = 0.03; Table 3). Moreover, in terms of diastolic dysfunction, 30% of the patients with HCM had grade II-III diastolic dysfunction, while 16% of patients with H-LVH had similargrades of diastolic dysfunction (p = 0.04). Higher grades of diastolicdysfunction accompanied by a greater ECV in both types of cardiomyopathies (p < 0.05), but no differences in native T1 or ECV were observed between the two diseases within the same grade of diastolic dysfunction (p > 0.05; Table 4). However, although the same range of ECV was found in the two groups (p = 0.6 for H-LVH vs. HCM), the LV mass/volume ratio was mildly impaired in H-LVH patients but was severely impaired in patients with HCM (1.18–1.66 of the 25–75^th^ percentile in the H-LVH group, 1.33–2.30 in the HCM group and 0.65–0.97 in the control group, p < 0.001; Fig. 2a).

**Table 3:** ECV and Native T1 Distribution in Patients with Normal Diastolic Function.

	Control	H-LVH	HCM	P value*
Patient Number (%)	35 (100%)	13 (30%)^†^	22 (27%)^†^	<**0**.**001**
ECV (%)	24 ± 2	25 ± 3^†^	25 ± 2^†^	**0**.**03**
Native T1 myocardium, ms	1252 ± 49	1282 ± 64^†^	1288 ± 43^†^	**0**.**03**

^*^P value across the 3 groups.

^†^P < 0.05 indicates an individual group compared with the control group.

H-LVH = hypertension induced left ventricular hypertrophy; HCM = hypertrophic cardiomyopathy; and ECV = extracellular volume.

**Table 4:** ECV and Native T1 Distribution in Patients with Diastolic Dysfunction.

		Normal-Grade I Diastolic Function	Grade II-III Diastolic Dysfunction	P value*
Patient Number (%)	H-LVH	37 (84%)	7 (16%)	/
	HCM	57 (70%)	24 (30%)	/
	**P value**	/	**0**.**04**	/
ECV (%)	H-LVH	26 ± 3	30 ± 2	<**0**.**001**
	HCM	26 ± 2	29 ± 4	**0**.**001**
	**P value**	0.80	0.59	/
Native T1 myocardium, ms	H-LVH	1271 ± 85	1319 ± 55	0.23
	HCM	1300 ± 47	1324 ± 67	0.08
	**P value**	0.07	0.87	/

^*^P value across the 3 groups; ECV = extracellular volume.

In terms of systolic function, a hyperdynamic LVEF was observed in the HCM patients but not in patients with H-LVH (65.9–79.3% in the HCM group vs. 61.1–70.5% in the control group, p = 0.004; 57.6–74.9% in the H-LVH group vs. 61.1–70.5% in the control group, p = 0.7; Fig. 2b). The 95% CI of the mean showed similar results (p < 0.001 for the H-LVH group vs. the HCM group for the LV mass/volume ratio; p < 0.001 for the HCM group vs. the control group and p = 0.4 for the H-LVH group vs. the control group for LVEF).

**Figure 3:**
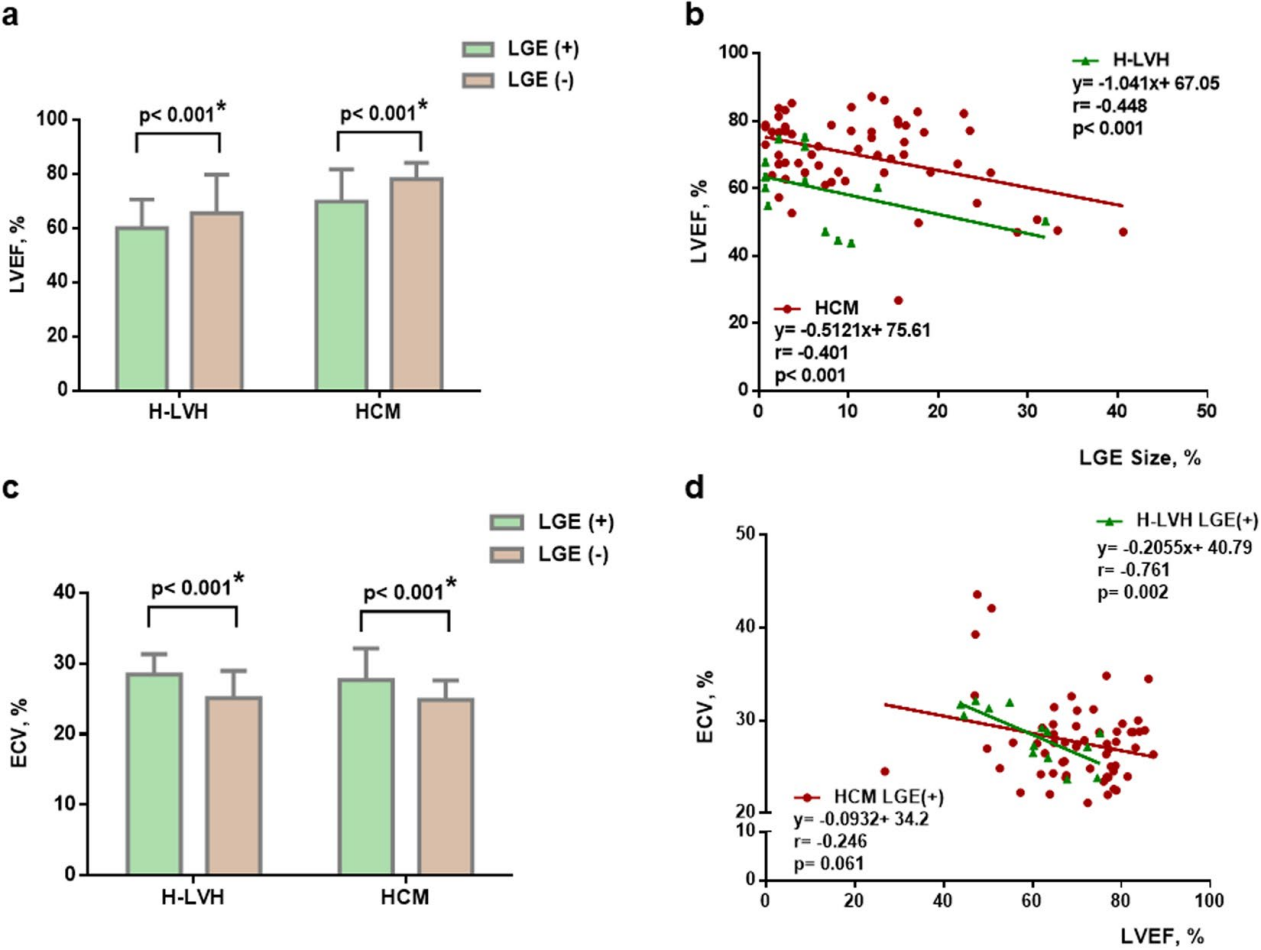
Relationship between LVEF and ECV in LGE (+) and LGE (−) subgroups. (**a**) LVEF in the LGE (+) or LGE (−) subgroups. (**b**) Relationship between LVEF and LGE size in patients with LGE (+) manifestations. (**c**) ECV in the LGE (+) or LGE (−) subgroups. (**d**) Relationship between ECV and LVEF in patients with LGE (+). ECV: extracellular volume; LVEF: left ventricular ejection fraction; HCM: hypertrophic cardiomyopathy; H-LVH: hypertension-induced left ventricular hypertrophy; LGE: late gadolinium enhancement.

To further explore the effect of pathological progression on left ventricular systolic function, we observed the relationship between regional (LGE) or diffuse myocardial fibrosis (ECV) and LVEF in the two diseases (Fig. 3). The LVEF was higher in patients without regional fibrosis (LGE−) than in those with regional fibrosis, independent of the aetiology of disease (LVEF in the LGE (+) vs. LGE (−) subgroups: 60.01 ± 10.65% vs. 65.55 ± 14.21% in the H-LVH group, p < 0.001; 69.85 ± 11.86% vs. 78.14 ± 6.08% in the HCM group, p < 0.001) (Fig. 3a). The LVEF decreased when the LGE size increased and the LVEF and LGE size were similar correlated in both the HCM and H-LVH groups (r = 0.4, respectively) (Fig. 3b). In terms of ECV, ECV was greater in patients without regional fibrosis (LGE-) than in those without regional fibrosis in both the H-LVH and HCM groups ((ECV value in the LGE (+) vs. LGE (−) subgroups: 28.46 ± 2.87% vs. 25.13 ± 3.86% in the H-LVH group, p < 0.001; 27.69 ± 4.5% vs. 24.85 ± 2.81% in the HCM group, p < 0.001)) (Fig. 3c). Interestingly, ECV was negatively correlated with LVEF in H-LVH (r = −0.761, p = 0.002), while no significant linear correlation was observed in HCM group (r = −0.246, p = 0.06) (Fig. 3d).

## Discussion

The main findings of this study are summarized here. An increased ECV was observed in patients with HCM, even in the sub-group of patients with normal diastolic function. An increased ECV was associated with a severely impaired LV mass/volume ratio and a hyperdynamic LVEF in the HCM group, but it was associated with only a mild impairment of the LV mass/volume ratio and was not associated with hyperdynamic LVEF in the H-LVH group. Taking both LGE and ECV into account might be a reasonable way to differentiate between HCM and H-LVH.

Unlike single native T1 or post-contrast T1 measurements alone, ECV, which is a biopsy- validated surrogate measure of interstitial fibrosis^10^, can be noninvasivelydetermined using CMR without impacting the field strength, gadolinium clearance rate, time of measurement, injection dose, body composition, or haematocrit, which ware limitations of single native T1 or post-contrast T1 measurements^11^. Clinically, increases in ECV have been reported in a variety of cardiac diseases, including myocardial infarction, myocarditis, HCM and H-LVH^9^. From a prognostic perspective, increased ECV or scarring may be related to ventricular tachyarrhythmia^12^. In the current study, although the maximum end diastolic wall thickness in the HCM group was significantly thicker than that of the H- LVH group, the overall LVM and LVMI were similar between the disease groups. The ECV was also increased in both groups, which is consistent with previous publications^13^, but the increase was not specific to either disease and the same LVMI corresponded to a similar range of ECVs.

In terms of cardiac function, ECV is a relatively stable indicator of the presence of diastolic dysfunction^3^. In young patients with aortic stenosis, the myocardial ECV is significantly increased and is associated with echocardiographic indices of diastolic dysfunction. Additionally, an increased ECV arises from a reduced rate of relaxation and increased stiffness^14^. In our current study, we used both myocardial stiffness indices (the LV mass/volume ratio) and combined-parameter-determined diastolic grading to assess diastolic function. To our surprise, an increased ECV was observed in both cardiomyopathies (nearly 30% of each group), even in patients with normal diastolic function, which promoted us to suggest that ECV may serve as an early indicator of diastolic impairment susceptibility from a mechanical aspect. Theoretically, interstitial fibrosis occurs earlier and is followed by clinical manifestations, including myocardial stiffness or a decreased LVEF. An increased ECV was associated with a severely impaired LV mass/volume ratio and was observed in 30% of the patients with grade II-III diastolic dysfunction in the HCM group. However, in the H-LVH group, it was associated with a mild impairment of the LV mass/volume ratio and showed a much weaker relationship to moderate-to-severe diastolic impairments. The mechanisms underlying this phenomenon are at least partially due to extracellular matrix distribution. Despite similar ECV values, the distribution of the extracellular matrix was distinct between the two disorders (Fig. 1). The homogeneous abnormalities in the myocardium in H-LVH patients make the LGE more difficult to visually recognize, while much denser but concentrated foci are observed in HCM patients. Histologically, hypertensive cardiac remodelling is a reactive myocardial fibrosis process that extends from the perivascular space into the intermuscular interstitium^1^. LVH involves adaptive changes to pressure overload. Fibrotic changes occur gradually according to changes in pressure overload, which are usually mild but are also extensive. However, the characteristics of HCM include structurally altered arterioles with unevenly distributed para-vascular fibrosis found histologically^2,15^, which may account for the patchy fibrotic cardiomyopathy process. The mass/volume ratio may be explained by increases in the LV mass and decreases in the volume. However, the LV mass, but not the volume, was increased in the HCM group, leading to a more severely increased LV mass/volume ratio that indicated the marked presence of diastolic dysfunction in the HCM group even though the range of ECV was similar to that in the H-LVH group.

In terms of systolic function, the LVEF may theoretically increase in response to increases in LVM. Interestingly, this comprehensive response was only observed in the HCM group and not in the H-LVH group. We postulated that there are two explanations for this observation. First, the LVM was correlated with increased ECV in the HCM group, which may have affected the myocardial strain in a specific pattern. The absolute global longitudinal strain (GLS) values in all the endocardial and epicardial myocardial layers were significantly lower in HCM subjects than in controls. However, to maintain the LVEF, the endocardial global circumferential strain (GCS) was maintained to compensate for the decrease in endocardial GLS. Therefore, the ratio of endocardial strain to epicardial strain may increase to achieve a compensatory effect^16^. Second, the hyperdynamic LVEF phenomenon had passed the hyperdynamic stage, which was perhaps due to the timing of the observation of the LVEF in the H-LVH group. Ozawa *et al*. suggested that a larger LV size corresponds to a smaller compensatory effect^16^, suggesting that patients with H-LVH need more extensive evaluations and antihypertensive treatments before tissue remodelling and systolic decompensation occur.

To further explore the effect of pathological progression on left ventricular function, we combined LGE and ECV to evaluate the relationship between regional or diffuse myocardial fibrosis and LVEF in these two diseases. We found that taking both LGE and ECV into account might be an option to differentiate HCM and H-LVH; howeve, this finding needs further validation in larger studies.

Limitations: First, there was a large overlap in ECV between HCM and H-LVH. ECV represents the extent of fibrosis in both diseases. Therefore, ECV can neither serve as a predictor of ongoing hypertrophic progression nor provide aetiologicinformation. Second, this study was conducted with a relatively small sample size in the H-LVH group because only patients with a definite elevation in LVM were recruited. Third, the ability of ECV to quantify diffuse fibrosis can only be interpreted on a group basis (rather than on a per-patient basis) due to partial volume effects, patient motion, etc. Combining ECV images and LGE may increase diagnostic confidence because the LGE image quality is generally excellent and does not have the same artefacts. Finally, the assessment of diastolic function in this study was based on echocardiographic measurements, which may not always perfectly agree with invasive assessments.

Conclusions: Distinct extracellular matrix distributions accounted for the clinical imaging manifestations of H-LVH and HCM. An increased ECV was found in patients with the cardiomyopathies with normal diastolic function. Further exploration of the prognostic value of ECV is warranted. Myocardial ECV was similarly increased in the H-LVH and HCM groups and was differentially associated with systolic and diastolic function. Taking both LGE and ECV into account might be a reasonable way to differentiate between HCM and H-LVH.

Perspectives: The clinical implications of this study include the following: the future exploration of the prognostic value of the ECV; the use of ECV in HCM patients with normal diastolic function to screen for subjects with increased myocardial fibrosis and observe the clinical significance of this detection; and the increasing importance of ECV findings for pharmaceutical treatment timing and treatment options in these disorders. Second, based on the differences in systolic and diastolic manifestations between these two cardiomyopathies, more prospective studies should focus on combining systolic and diastolic functional measures with other parameters, such as wall thickness, myocardial deformation and LGE, to further contribute to differentiating between HCM and H-LVH.

## Materials and Methods

### Patient Enrolment

All participants provided written informed consent and the protocol was approved by the IRB. All experiments were performed in accordance with the current imaging guidelines and regulations. Consecutive subjects were prospectively enrolled into 3 cohorts between July 2014 and February 2016. The cohorts were as follows: an H-LVH group, an HCM group and a control group.

The diagnosis of HCM was supported by left ventricular hypertrophy diagnosed on echocardiography (LVH; wall thickness >15 mm) with either a genetic determination of a pathogenic mutation or a history of syncope with systolic anterior motion of the mitral valve, mitral regurgitation and resting left ventricular outflow tract obstruction.

The diagnosis of LVH was based on medical history and conventional echocardiography, including long durations of uncontrolled hypertension, i.e., for at least 5 years, with a systolic blood pressure (BP) ≥150 mm Hg, diastolic BP ≥90 mm Hg, or both in the absence of other cardiac or systemic diseases and echocardiographic demonstration of a hypertrophic LV (maximal LV wall thickness >11 mm). Subjects were enrolled in the H-LVH group if their left ventricular mass (LVM) by body surface area (LVMI), as measured by cardiac magnetic resonance imaging, was >81 g/m^2^ for men or >61 g/m^2^ in women, as defined by Olivotto *et al.*^17^.

Candidates who served as controls were used to establish baseline myocardial T1 and ECV values. The controls were generally scheduled for CMR for atypical chest pains, palpitations, or pre-operative evaluation for non-cardiac surgery and had no known hypertension, other systemic illness or family history of cardiomyopathy or sudden death. They also had normal electrocardiographic, echocardiographic, or CMR results, including a normal wall thickness and LVMI without hyperenhancement.

The exclusion criteria were age <18 years old or >80 years old, documented coronary artery diseases or prior angiography for coronary artery disease (>50% stenosis), Framingham risk level ≥5% in the previous 10 years, or atrial fibrillation during echocardiography, which impedes the grading of diastolic function. Patients with known infiltrative cardiomyopathies or septal ablations for drug-refractory hypertrophic obstructive cardiomyopathy or patients with standard metallic contraindications for CMR were also excluded.

### Echocardiographic Assessment of Diastolic Function

The assessment of LV diastolic function was an integral part of routine echocardiographic examinations (E9, GE Healthcare, Milwaukee, WI, USA). The mitral E/A ratio, tricuspid regurgitation velocity, mitral DT, septal E′ and lateral E′ were recorded. The left atrial volume was obtained using the apical 4-chamber and 2-chamber views. The evaluation of left ventricular diastolic function was performed based on the 2016 recommendations for the evaluation of left ventricular diastolic function from the American Society of Echocardiography and the European Association of Cardiovascular Imaging^18^. Normal values for age-related Doppler-derived diastolic measurements for Chinese patients were adapted from the EMINCA study^19^.

### CMR Protocol

All CMR examinations were performed using a Philips 3 T Ingenia MR system (Philips Healthcare, Best, Netherlands).

#### Cardiac Function

A volumetric cavity assessment was obtained by whole-heart coverage of short-axis slices (7- mm thick with a 3-mm gap). Additionally, video images of 3 long-axis views (4-chamber, 2- chamber and 3-chamber views) were acquired. All video images were acquired using a balanced, steady-state, free precession sequence combined with parallel imaging (SENSE Sensitivity Encoding, factor 2) and retrospective gating during a gentle, expiratory breath-hold (echo time [TE]/repetition time [TR]/flip-angle: 1.5 ms/3.0 ms/45° and spatial resolution 1.9 × 2.1 × 7 mm).

#### Native and Post-Contrast T1 Mapping

We integrated native and post-contrast myocardial T1 mapping into our routine imaging protocol to determine the ECV. A steady-state free precession, single breath-hold modified Look-Locker inversion recovery sequence (Philips MR research device, “MOLLI R5.1”) was used for T1 mapping and a “5–3–3” scheme (in seconds) was chosen to reduce respiratory motion artefacts, contractile motion blurring and black banding artefacts. The scheme was performed on an equatorial 3 end-diastolic LV short-axis slice (basal, mid-ventricle and apex) before and 15 min after a bolus intravenous injection of 0.15 mmol/kg of gadopentetate dimeglumine (Gd-DTPA; Magnevist, Bayer Healthcare, Berlin, Germany). The typical acquisition parameters were as follows: field of view, 320 × 320; TR, 2.3 ms; TE 1.06, ms; flip angle, 20°; interpolated voxel size, 2.0 × 2.0 × 8 mm; 160 phase-encoding steps; SENSE = 2; cardiac delay time, TD = 350 ms; and 1,200-ms acquisition time for a single image. The region of interest was positioned at the magnet isocenter. Shimming and centre frequency adjustments were performed to generate images free from off-resonance artefacts if necessary.

#### Evaluation of Late Gadolinium Enhancement

LGE imaging was performed with gapless whole-heart coverage of short axis slices 10 min after the administration of a dose of 0.15 mmol/kg body weight of gadopentetate dimeglumine using a mid-diastolic inversion-prepared, 2-dimensional gradient echo sequence (TE/TR/flip: 1.7 ms/3.3 ms/25°, interpolated voxel size 1.6 × 1.9 × 10 mm). Segmented LGE images with at least three matching slices and native T1 images were also acquired.

### Image Analysis

#### LV Morphology and Function

Routine CMR analysis was performed using commercially available software (View-Forum, Extended Workspace, Philips Healthcare). The CMR data analysis was performed by 2 observers who had experience with CMR and who were blinded to the clinical information. Systolic function was represented by the left ventricular ejection fraction (LVEF). Because the LV mass/volume ratio is closely related to the LV flow propagation velocity and early intraventricular pressure gradient, the LV mass/volume ratio was adopted as a measure of LV stiffness with a normal range from 1–1.5^3,20,21^.

For LV volume and mass, endocardial LV borders were manually traced at end-diastole and end-systole. The papillary muscles were included as part of the LV cavity volume. LV end-diastolic and end-systolic volumes were determined using Simpson’s rule. All volumetric indices were normalized to the body surface area.

The LGE images were examined for the presence and extent of regional fibrosis of the LV (cvi^42^ Version 5.5.6.1, Alberta, Canada).

#### ECV Quantification

MRmap software was used to create parametric maps of the MR relaxation times (IDL 8.5, ITT Visual Information Solutions, Boulder CO, USA)^22^. The maps were calculated pixel by pixel according to the selection of the user and native and post-contrast T1 values were measured in the myocardium and blood pool. All three slices from base to apex, before and after contrast, were measured. The region of interest was placed on the 16 segments of the myocardium without avoiding the LGE scar. Care was taken to avoid “contamination” from the signal from the blood pool.

Reported T1 values were derived with the operator blinded to the LGE image groups. In addition to the T1 values of the myocardium and blood pool, we calculated the ECV according to the formula: ECV = λ * (1 − haematocrit), where λ = [ΔR1 myocardium]/[ΔR1 blood pool] before and after gadolinium contrast injection (where R1 = 1/T1). Haematocrit was tested on the same day in all subjects.

#### Quality Assessment of T1 Maps

The “5-3-3” scheme (in seconds) from MOLLI 5.1 is independent of heart rate and already compensated for respiratory motion artefacts, contractile motion blurring and black banding artefacts. The position of the source images was initially manually adjusted using MRmap to correct for potential mis-registration caused by translational displacement in cases of poor breathholding^22^. T1 maps were then scored for overall quality based on a 5-point scale^23^. Briefly, a score of 1 (non-diagnostic) indicated that the T1 value could not be reliably measured anywhere in the myocardium and the patient was then excluded from the current study. Score of 2, 3, 4 and 5 indicated poor, fair, good and excellent performance, respectively, providing the ability to distinguish regions of normal myocardium from noise or artefacts.

Moreover, a second blinded operator analysed more than 50% of the cases, which were randomly selected. The same analytic technique was used and the results were compared using the Bland-Altman method.

### Statistical Analysis

Quantitative data are presented as the mean ± SD or the median (interquartile range). Categorical data are presented as numbers and percentages. All quantitative data were assessed for normality using the Kolmogorov-Smirnov test and comparisons among the 3 groups were performed with nonparametric Kruskal-Wallis and Dunnett’s tests. For the qualitative data, comparisons of the three groups were performed using Pearson’s chi-squared test or Fisher’s exact test. Inter-observer reproducibility of T1 mapping and ECV measurements were assessed using the Bland- Altman method and an intra-class coefficient analysis. All analyses were performed using IBM SPSS Statistics software, version 17.0 (© 2010 SPSS, Inc.) Two-tailed p-values < 0.05 were considered significant.

### Availability of data and material

The datasets used and/or analysed during the current study are available from the corresponding author on reasonable request.

### Data availability statement

Supporting data are available to editorial board members and referees.

### Ethics Approval and Consent to Participate

The IRB of Renji Hospital approved the protocol.

### Consent for Publication

All participants provided written informed consent.

## References

[1] Diez J (2007). Mechanisms of cardiac fibrosis in hypertension. Journal of clinical hypertension.

[2] Maron BJ, Wolfson JK, Epstein SE, Roberts WC (1986). Intramural (“small vessel”) coronary artery disease in hypertrophic cardiomyopathy. Journal of the American College of Cardiology.

[3] Gaasch WH, Aurigemma GP (2015). CMR imaging of extracellular volume and myocardial strain in hypertensive heart disease. JACC. Cardiovascular imaging.

[4] Puntmann VO (2013). Native T1 mapping in differentiation of normal myocardium from diffuse disease in hypertrophic and dilated cardiomyopathy. JACC. Cardiovascular imaging.

[5] Coelho-Filho OR (2014). Cardiac magnetic resonance assessment of interstitial myocardial fibrosis and cardiomyocyte hypertrophy in hypertensive mice treated with spironolactone. Journal of the American Heart Association.

[6] Coppini R (2013). Late sodium current inhibition reverses electromechanical dysfunction inhuman hypertrophic cardiomyopathy. Circulation.

[7] Kellman P (2012). Extracellular volume fraction mapping in the myocardium, part 2: initial clinical experience. Journal of cardiovascular magnetic resonance: official journal of the Society for Cardiovascular Magnetic Resonance.

[8] White SK, Sado DM, Flett AS, Moon JC (2012). Characterising the myocardial interstitial space: the clinical relevance of non-invasive imaging. Heart.

[9] Kuruvilla S (2015). Increased extracellular volume and altered mechanics are associated with LVH in hypertensive heart disease, not hypertension alone. JACC. Cardiovascular imaging.

[10] Flett AS (2010). Equilibrium contrast cardiovascular magnetic resonance for the measurement of diffuse myocardial fibrosis: preliminary validation in humans. Circulation.

[11] Mewton N, Liu CY, Croisille P, Bluemke D, Lima JA (2011). Assessment of myocardial fibrosis with cardiovascular magnetic resonance. Journal of the American College of Cardiology.

[12] Amano Y (2014). Delayed enhancement magnetic resonance imaging in hypertrophic cardiomyopathy with Basal septal hypertrophy and preserved ejection fraction: relationship with ventricular tachyarrhythmia. Journal of computer assisted tomography.

[13] Brouwer WP (2014). *In-vivo* T1 cardiovascular magnetic resonance study of diffuse myocardial fibrosis in hypertrophic cardiomyopathy. Journal of cardiovascular magnetic resonance: official journal of the Society for Cardiovascular Magnetic Resonance.

[14] Hutchinson KR, Lord CK, West TA, Stewart JA (2013). Cardiac fibroblast- dependent extracellular matrix accumulation is associated with diastolic stiffness in type 2 diabetes. PloS one.

[15] Calhoun DA (2011). Effects of a novel aldosterone synthase inhibitor for treatment of primary hypertension: results of a randomized, double-blind, placebo- and active- controlled phase 2 trial. Circulation.

[16] Ozawa K (2015). Characteristic myocardial strain identified in hypertrophic cardiomyopathy subjects with preserved left ventricular ejection fraction using a novel multi-layer transthoracic echocardiography technique. International journal of cardiology.

[17] Olivotto I (2008). Assessment and significance of left ventricular mass by cardiovascular magnetic resonance in hypertrophic cardiomyopathy. Journal of the American College of Cardiology.

[18] Nagueh SF (2016). Recommendations for the Evaluation of Left Ventricular Diastolic Functionby Echocardiography: An Update from the American Society of Echocardiography and the European Association of Cardiovascular Imaging. Journal of the American Society of Echocardiography: official publication of the American Society of Echocardiography.

[19] Yao GH (2016). Doppler Echocardiographic Measurements in Normal Chinese Adults (EMINCA): a prospective, nationwide, and multicentre study. European heart journal cardiovascular Imaging.

[20] Buakhamsri A (2009). Impact of left ventricular volume/mass ratio on diastolic function. European heart journal.

[21] Gaasch WH, Zile MR (2011). Left ventricular structural remodeling in health and disease: with special emphasis on volume, mass, and geometry. Journal of the American College of Cardiology.

[22] Messroghli DR (2010). An open-source software tool for the generation of relaxation time maps in magnetic resonance imaging. BMC medical imaging.

[23] Kellman P, Wilson JR, Xue H, Ugander M, Arai AE (2012). Extracellular volume fraction mapping in the myocardium, part 1: evaluation of an automated method. Journal of cardiovascular magnetic resonance: official journal of the Society for Cardiovascular Magnetic Resonance.

