# Author Correction: The Significance of Interstitial Fibrosis on Left Ventricular Function in Hypertensive versus Hypertrophic Cardiomyopathy

**DOI:** 10.1038/s41598-018-30170-w

**Published:** 2018-08-10

**Authors:** Meng Jiang, Zi Wang, Xuan Su, Xingrong Gong, Jun Pu, Lianming Wu, Chang Liu, Qiuying Yao, Lingcong Kong, Jianrong Xu, Ben He

**Affiliations:** 1 Department of Cardiology, Renji Hospital, School of Medicine Shanghai Jiaotong University, Shanghai, 200127 China; 2 Department of Radiology, Renji Hospital, School of Medicine Shanghai Jiaotong University, Shanghai, 200127 China; 3 Department of Epidemiology and Biostatistics, West China School of Public Health, Sichuan University, Chengdu, 610041 China

Correction to: *Scientific Reports* 10.1038/s41598-018-27049-1, published online 03 July 2018

In the original version of this Article, Lingcong Kong was incorrectly listed as a corresponding author. The correct corresponding authors for this Article are Jianrong Xu and Ben He. Correspondence and request for materials should be addressed to xujrdoc10@163.com and heben_rj@163.com. This error has now been corrected in the HTML and PDF versions of the Article.

